# Aerodynamic Characteristics of a Tandem Flapping Wing in Inclined Stroke Plane Hovering with Ground Effect

**DOI:** 10.3390/biomimetics10040212

**Published:** 2025-03-30

**Authors:** Arun Raj Shanmugam, Chang Hyun Sohn, Ki Sun Park

**Affiliations:** 1 Department of Mechanical and Aerospace Engineering, United Arab Emirates University, Al Ain 15551, United Arab Emirates; arunraj.v2009@gmail.com; 2 School of Mechanical Engineering, Kyungpook National University, Daegu 41566, Republic of Korea; chsohn@knu.ac.kr

**Keywords:** aerodynamics, dragonflies, flapping wing, ground effect, hovering

## Abstract

The present two-dimensional study investigates the ground effect on the aerodynamic characteristics of a tandem flapping wing in inclined stroke plane hovering using ANSYS Fluent. The role of various wing kinematics parameters (flapping frequency f, stroke amplitude A_o_/c, and phase difference ψ = 0° and 180°), in combination with ground distance (D* = D/c), is studied. The results reveal that a large stroke amplitude A_o_/c decreases vertical force generation for both in-phase and counter-stroking patterns. The vertical force notably increases for both in-phase and counter-stroking wings when D* is extremely small (D* = 0.5). A maximum vertical force enhancement of approximately 65% and 35% is observed for in-phase and counter-stroking patterns, respectively, at D* = 0.5. This enhancement is primarily attributed to the strengthening of detached vortices on the lower surface of the wings during the middle of the downstroke when flapping at extremely small ground distances. In addition, the wing–wing interaction and secondary rebound vortex, caused by wing–ground interaction, also play a key role in vertical force generation. The wing–ground interaction positively influences both vertical and thrust force generation for in-phase and counter-stroking wings at small ground distances. In general, the vertical and thrust forces generated by in-phase stroking wings are greater than those produced by counter-stroking wings.

## 1. Introduction

Natural flyers and swimmers adopt several strategies, such as schooling [1], drafting [2], formation flight [3], and ground effect [4,5,6], to enhance their locomotive performance. Among these, the ground effect, utilized by natural flyers, has garnered renewed attention from researchers due to its potential applications in micro-aerial vehicle (MAV) technology. Ground effect, commonly known as “wing–ground interaction”, refers to the increase in force coefficients when the distance between the wing and the ground is less than or equal to one wingspan [4]. For real-life applications, various parameters, including the Reynolds number (Re), Strouhal number (St), and atmospheric turbulence, in addition to ground proximity, can affect the aerodynamic characteristics of a wing in ground effect.

Flapping flight, in general, is more aerodynamically efficient than fixed-wing flight and offers greater maneuverability [7]. For this reason, researchers have looked to natural flyers for inspiration. Dragonflies are well known for their exceptional predatory abilities, agility, and maneuverability. They typically operate in a low Reynolds number regime of 100–10,000 by flapping their wings at a stroke plane inclination (β) of 60° and a stroke amplitude (A_o_/c) of 2.5–5c [8,9]. The lower and upper limits of flapping frequency (f) are 20 Hz and 157 Hz, respectively [10,11].

In the past, experimental and numerical studies have been conducted to uncover the secrets of dragonfly flight. Some studies have explored the wing kinematics of dragonflies using a single-wing arrangement without considering the interaction between the forewing and hindwing [9,12,13,14,15,16]. Wang [9] adopted a numerical approach and found that dragonflies use a drag-based strategy to generate aerodynamic forces, with nearly three-quarters of their weight balanced by drag. Other studies have investigated the effects of forewing–hindwing phase differences [17,18,19,20]. Three common stroking patterns in dragonflies are in-phase stroking, counter-stroking, and phased stroking. In-phase stroking has been found to generate the highest average vertical force coefficient, whereas counter-stroking minimizes power consumption while producing the required average vertical force coefficient [17,18,19,20]. Meanwhile, some experimental studies have examined the unsteady force-generation mechanisms of dragonflies. Key mechanisms, including delayed stall, wake capture, rotational lift, and forewing–hindwing interaction, have been identified as significant contributors to force generation [21,22].

The complex interaction between a MAV and the ground presents a challenging problem in aerodynamics [23]. Although wing–ground interaction can provide additional lift, it can also introduce difficulties in MAV control [24]. Rayner [4] developed a theory using a lifting-line wing model to predict the effects of ground proximity on animal flight performance. It was found that ground effect reduces the required power by approximately 35%. Gao and Lu [25] investigated the ground effect on a hovering wing using numerical simulations, identifying three force behavior kinds: force enhancement, reduction, and recovery. The interaction between existing vortices and the wing increases the strength of the leading-edge vortex (LEV), resulting in vertical force enhancement. Lu et al. [26] examined the ground effect on a fruit-fly-inspired hovering wing and found that the increase in force coefficients for D* < 2 may be attributed to strong wing-wake interaction. Extending this work, Lu et al. [27] determined that LEV attachment plays a vital role in force generation when the wing is close to the ground.

Meanwhile, Wu et al. [28] investigated the ground effect on a plunging and pitching flapping wing in forward flight using the immersed boundary-lattice Boltzmann method (IB-LBM). The effects of two parameters, namely ground distance and flapping frequency, were studied. At low flapping frequencies, both the average vertical and horizontal force coefficients increased. In contrast, at high flapping frequencies, the average vertical force coefficient increased, while the horizontal force coefficient decreased. Wu et al. [29] investigated the ground effect on a plunging and pitching flapping wing, examining the effects of three parameters: mean distance, amplitude, and frequency. They found that ground effect improves power extraction efficiency by up to 28.6%. In a later study, Wu et al. [30] determined that the maximum power extraction for a plunging and pitching flapping wing in ground effect occurs at a reduced frequency of 0.2. Mivehchi et al. [31] investigated the ground effect on a heaving and pitching flapping wing using both experimental and numerical approaches, finding that propulsion efficiency increases slightly when the ground distance is small.

Dragonflies exhibit remarkable flight capabilities. They possess two sets of forewings and hindwings that can function independently to modify their motion. Srinidhi and Vengadesan [32] investigated the ground effect on a hovering dragonfly-inspired wing at Re = 100. They found that force generation is influenced by vortices rebounding from the ground due to changes in the effective angle of attack experienced by the wings. Additionally, they revealed that the influence of rebound vortices is stronger when the forewing–hindwing spacing is small. Abdizadeh et al. [33] investigated the ground effect on dragonfly-inspired flapping wings in forward flight. Two types of wing shapes, NACA4412 and corrugated dragonfly-inspired, were studied. They found that the lift-to-drag ratio of the wing is significantly affected by the ground effect. The corrugated wing has a better lift-to-drag ratio than the NACA4412 at Re = 5000, but its performance drops when the Reynolds number is increased to 50,000.

Recently, the ground effect has been studied for various engineering applications, such as flapping wing energy harvesters [34,35,36,37] and unmanned underwater vehicles [38]. Li et al. [38] explored the hydrodynamic characteristics of ground effect on oscillating wings in terms of propulsive performance. They found that the ground effect significantly impacts the propulsive performance of oscillating wings. At a low Strouhal (St) number, a sharp increase in thrust force with ground distance is observed, while the lift force increases steadily. However, at a high St number, the lift force initially increases with ground distance and then decreases.

The new-generation MAV is designed to navigate indoor environments and tight spaces. The performance of MAVs can be strongly influenced by the presence of the ground, especially during hovering, take-off, and landing. Only a few studies have investigated the significance of ground effect on the aerodynamics of tandem flapping wings [32,33]. Srinidhi and Vengadesan [32] studied tandem flapping wings in an inclined stroke plane hovering using a similar wing kinematics. Meanwhile, Abdizadeh et al. [33] studied a flapping wing in a 90° stroke plane during forward flight. However, the role of various parameters of wing kinematics in inclined stroke plane hovering, such as flapping frequency (f), stroke amplitude (A_o_/c), forewing–hindwing phase difference, and Reynolds number (Re), is not yet fully understood for tandem flapping wings in ground effect.

Against this backdrop, the present numerical study consisting of 50 simulation cases is performed. For such a parametric investigation study, two-dimensional simulations are preferred over three-dimensional ones due to computational cost constraints. While the three-dimensional simulations are significant in capturing full aerodynamic behavior, such simulations are less suitable for extensive parametric studies. Furthermore, many past studies on inclined stroke plane hovering have demonstrated that the two-dimensional model can effectively predict key flow features responsible for aerodynamic force generation with reasonable accuracy [9,12,14,15,16,17,18]. Therefore, in this study, a two-dimensional numerical analysis is performed to determine the aerodynamic characteristics of a tandem flapping wing in inclined stroke plane hovering near the ground using ANSYS Fluent V18. Exploring the aerodynamics of ground effect in inclined stroke plane hovering is valuable for the design of MAVs. The knowledge gained from this study can be applied to enhance the performance, efficiency, and capabilities of modern insect-size MAVs.

## 2. Numerical Method and Validation

This work investigates the aerodynamics of a tandem flapping wing in inclined stroke plane hovering. The inclined stroke plane hovering is observed in insects like dragonflies. Inspired from dragonflies, a stroke plane inclination (β) of 60° and the flapping frequency (f) range of 26–157 Hz is used [10,11]. Meanwhile, the stroke amplitude (A_o_/c) in inclined stroke plane hovering for the 2D wing kinematics typically varies in the range of 2.5 to 5 [8,9]. The wing motion parameters used in this work are shown in Table 1. In the present study, the dependence of aerodynamic force generation on viscosity is investigated while keeping A_o_/c = 2.5 and f = 26 Hz, following Wang’s study [39]. Three different viscosities and their respective Reynolds numbers are specified in Table 1. The wing spacing L is measured horizontally between the two-stroke planes of the forewing and hindwing.

Three distinct flapping wing patterns are often observed in dragonfly flight: in-phase stroking (ψ = 0°), counter-stroking (ψ = 180°), and phased-stroking, each influenced by phase shift. In in-phase stroking, tandem wings flap together simultaneously, generating stable vertical force to counter weight. In counter stroking, the wings beat out of phase, creating complex aerodynamic interaction between the wings that improve maneuverability and stability for rapid and precise flight control. Phased-stroking generates more horizontal force while reducing vertical force, beneficial for forward flights [40]. Since the study primarily focuses on hovering flight, where vertical force generation is crucial to counteracting weight, only in-phase stroking and counter-stroking are examined here.

**Figure 1:**
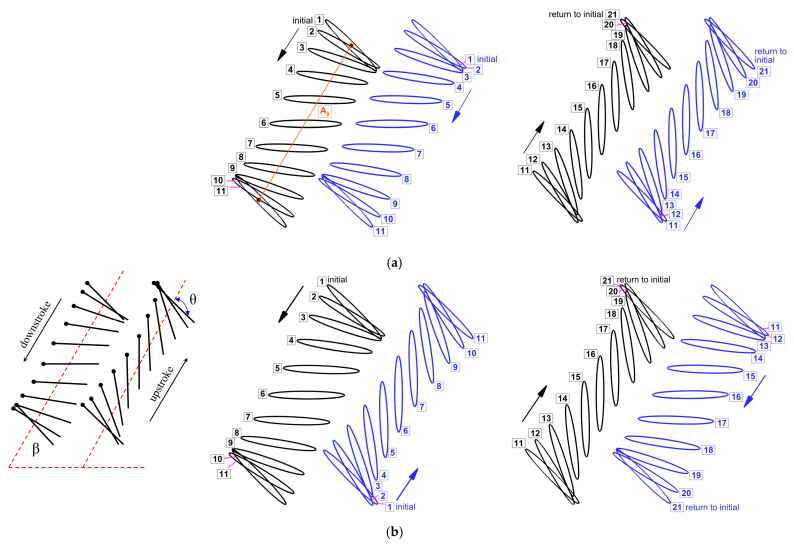
Wing kinematics of a tandem flapping wing in inclined stroke plane. (**a**) In-phase stroking wing (ψ = 0°). First half of the time period (**on the left**) and second half of the time period (**on the right**). (**b**) Counter stroking wing (ψ = 180°). First half of the time period (**on the left**) and second half of the time period (**on the right**). (θ indicates rotational angle). (arrow shows up/down stroke).

For 2D simulations, the wing kinematics of tandem flapping wing in inclined stroke plane hovering can be defined using simplified motions, namely heaving [x(t), y(t)] and pitching α(t). The flapping wing motion of a tandem wing in inclined-stroke-plane hovering is illustrated in Figure 1. The in-phase stroking tandem wing motion is shown in Figure 1a, whereas the counter-stroking motion is shown in Figure 1b. The flapping wing motion is divided into multiple stages in both downstroke and upstroke, with corresponding numerical labels, as shown in Figure 1. The time instant t/T and angle of rotation at each stage is presented in Table 2 to enable better analysis on time histories of aerodynamic force generation and vortex evolution.

The pitching and heaving motions of forewing and hindwing in linear trajectory can be written in the form of Equations (1)–(6). This model, originally proposed by Wang [9], represents one of the simplest forms of hovering motion, but it enables the dependence of force generation and flow dynamics across various styles of hovering that are observed in dragonflies (β = 60°) and fruit flies (β = 0°). Accurately replicating the precise wing motion of real dragonflies would require highly complex trigonometric functions, making them less suitable for the numerical study. Therefore, several assumptions are made, consistent with previous studies on tandem flapping wings in inclined stroke plane hovering [9,12,14,17,32]. The assumptions made in the wing kinematics are as follows:

1. The flapping wing trajectory is assumed to be linear, whereas real dragonflies exhibit a complex figure-eight motion, with the upstroke and downstroke motions following different figure-eight trajectory.
2. The downstroke and upstroke durations are assumed equal, while in reality, the downstroke is significantly longer than the upstroke.
3. The pitch motion is modeled as a continuous sinusoidal function. However, in real dragonflies, the pronation and supination phases are relatively shorter than the translation phase, which is not considered in the wing kinematics.
4. The wings are treated as rigid, without considering flexibility or twisting.

This wing kinematics model, however, effectively captures key flow features and predict aerodynamic forces with reasonable accuracy. As concluded by Kim and Choi [41], the adopted wing kinematics is reliable and effective for predicting aerodynamic characteristics in inclined stroke plane hovering observed in insects like dragonflies.

The forewing–hindwing phase difference (ψ) is set according to the flapping pattern. For an in-phase stroking wing (ψ = 0°), the first half of a time period (or cycle) is a downstroke, and the second half is an upstroke for both the forewing and the hindwing, as shown in Figure 1a. On the contrary, for a counter stroking wing (ψ = 180°), the first half of a time period (or cycle) is a downstroke, and the second half is an upstroke for the forewing. However, for the hindwing, the first half is an upstroke, and the second half is a downstroke, as shown in Figure 1b.

(1) $$x_{f}\left(t\right)=\frac{A_{o}}{2}\;\cos\left(\beta_{f}\right)(1+\cos\left(\omega t\right))$$

(2) $$y_{f}\left(t\right)=\frac{A_{o}}{2}\;\sin\left(\beta_{f}\right)(1+\cos\left(\omega t\right))$$

(3) $$\alpha_{f}\left(t\right){=\alpha }_{o}-\;B\;\sin\left(\omega t+\varphi \right)$$

(4) $$x_{h}\left(t\right)=\frac{A_{o}}{2}\;\cos\left(\beta_{h}\right)(1+\cos\left(\omega t+\psi \right))$$

(5) $$y_{h}\left(t\right)=\frac{A_{o}}{2}\;\sin\left(\beta_{h}\right)(1+\cos\left(\omega t+\psi \right))$$

(6) $$\alpha_{h}\left(t\right){=\alpha }_{o}-\;B\;\sin\left(\omega t+\varphi +\psi \right)$$

where x(t) is the heave amplitude in the *x*-axis, y(t) is the heave amplitude in the *y*-axis, β is the stroke plane inclination, A_o_/c is the stroke amplitude in non-dimensional form, α(t) is the pitch amplitude about *z*-axis, B is the pitch amplitude, φ is the heave-pitch phase difference, α_o_ is the mean angle of attack, ψ is the forewing–hindwing phase difference, and ω = 2πf. Subscripts ‘f’ and ‘h’ indicates forewing and hindwing, respectively.

The Reynolds number Re is given by Equation (7).

(7) Re=ρ ue cμ

where ρ is the fluid density, c is the chord length, μ is the dynamic viscosity of fluid, $u_{e}$ is the effective velocity ($u_{e}{=u}_{\infty }{\;+\;u}_{\mathrm{heave}}$), $u_{\mathrm{heave}}$ is the maximum heaving velocity ($u_{\mathrm{heave}}{\;=\;\pi \;f\;A}_{o}$), and $u_{\infty }=0\;$for hovering flight.

The non-dimensional average vertical force coefficient $\bar{C_{v}}$ and average horizontal force coefficient $\bar{C_{h}}$ are given by Equations (8) and (9).

(8) $$\bar{C_{v}}=\frac{\int_{0}^{T}F_{v}\left(t\right)\;\mathrm{dt}}{0{.5\;\rho \;u}_{\mathrm{rms}\;}^{2}c\;T}$$

(9) $$\bar{C_{h}}=\frac{\int_{0}^{T}F_{h}\left(t\right)\;\mathrm{dt}}{0{.5\;\rho \;u}_{\mathrm{rms}\;}^{2}c\;T}$$

where $F_{v}\left(t\right)$ or $F_{v}$ is the instantaneous vertical force, $C_{v}\left(t\right)$ or${\;C}_{v}$ is the instantaneous vertical force coefficient, $F_{h}\left(t\right)$ or $F_{h}$ is the instantaneous horizontal force, $C_{h}\left(t\right)$ or${\;C}_{h}$ is the instantaneous horizontal force coefficient, and T is the time period. rms is the root mean square, and symbol ¯ indicates the time-average.

In this work, two enhancement factors, ξ and χ, are introduced to categorize the wing–ground interactions into beneficial or detrimental types. The definitions of ξ and χ are given by Equations (10) and (11). Since the wing–ground interaction effect is typically negligible for far-away ground distance cases (D* > 8), the $\bar{C_{v}}$ and $\bar{C_{h}}$ becomes stable beyond D* > 8. Therefore, the $\bar{C_{v}}$ and $\bar{C_{h}}$ predicted D* = 10 is used for the denominator in Equations (10) and (11) to estimate the enhancement factors ξ and χ. Finally, the ξ and χ are written as percentage increase or decrease for the sake of simplification.

(10) $$\xi =\frac{\bar{C_{v}}\;\left(\mathrm{with}\;\mathrm{wing}\;\mathrm{ground}\;\mathrm{interaction}\right)\;}{\bar{C_{v}}\;\left(\mathrm{without}\;\mathrm{wing}\;\mathrm{ground}\;\mathrm{interaction}\;\right)}$$

(11) $$\chi =\frac{\;\bar{C_{h}}\left(\mathrm{with}\;\mathrm{wing}\;\mathrm{ground}\;\mathrm{interaction}\right)}{\;\bar{C_{h}}\left(\mathrm{without}\;\mathrm{wing}\;\mathrm{ground}\;\mathrm{interaction}\;\right)}$$

**Figure 2:**
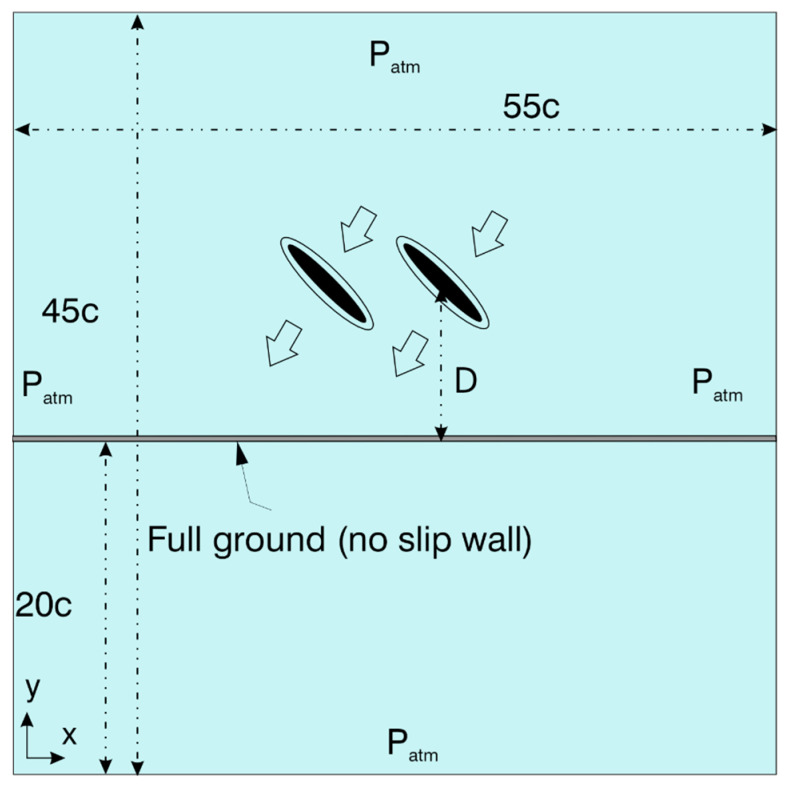
Schematic of the computational domain (not drawn to scale) and boundary conditions.

Figure 2 illustrates the computational domain and the boundary conditions used for performing the numerical simulations. The geometry of the flapping wing is an ellipse with a thickness-to-chord ratio of t/c = 10%, similar to the previous studies [12,14,17,32]. The flapping wing has a chord length c of 1 cm. Dragonflies, on the other hand, possess wings in the form of corrugated shapes, as indicated in the literature [33,42]. This is a reasonable assumption as the primary objective of the study is to explore the aerodynamics of flapping wings in close proximity to the ground for various wing kinematic parameters and Re. The wing material properties are not considered in the simulation as the flexibility is neglected.

The computational domain is a rectangle of length (l = 55c) and breadth (b = 45c) to ensure that the pressure outlet boundaries are far away from the wing (shown by inner filled ellipse). The domain comprises three zones, the forewing and hindwing zones (shown by outer ellipse) and the outer zone. Conformal interfaces are introduced in between them. The forewing and hindwing zones are discretized with triangular mesh cells, which do not deform with the wing motion, as shown in Figure 2. A total of 10 prism layers are created around the wing to capture the boundary layer effects. As the wing motion happens, the outer zone, discretized with triangular mesh cells, undergoes a smoothing and re-meshing operation, according to the dynamic mesh settings used in ANSYS Fluent. The spring-based smoothing method is used as it works better for the present model. The local re-meshing option is applied in the outer zone with a maximum skewness of 0.7 for the deformed mesh. A total of approximately 0.25 million cells are used to discretize the computational domain. The boundary conditions used for the numerical simulations are pressure outlet (atmospheric pressure P_atm_) for the outer boundaries, as shown in Figure 2, and no-slip wall condition for the wing and the ground.

The governing equations of the transient simulations are solved using a pressure-based solver with a finite volume-based software, ANSYS Fluent. The present study focuses exclusively on laminar flow, which is why turbulence modeling is not employed in the numerical model. The pressure implicit with split operator (PISO) algorithm is adopted for the pressure–velocity coupling. The standard scheme is used to discretize the pressure term, and the least square cell-based method is used to discretize the gradients. A second-order upwind scheme is employed to discretize the momentum equation. The transient formulation of the incompressible Navier-Stokes equation is achieved using a first-order implicit scheme.

A grid-independent test is carried out on the computational domain with the wing kinematics: A_o_/c = 2.5, β = 60°, α_o_ = 45°, B = 45°, φ = 0°, ψ = 0°, f = 26 Hz, L/c = 1.2, Re = 628, and D/c = 10. To find the optimum grid and time-step size, simulations are performed by simultaneously varying the number of grid cells and time-step sizes, as shown in Table 3. The instantaneous aerodynamic force coefficients become periodic after the 6^th^ cycle (time period) for all the simulated cases. Therefore, the force coefficients of the 10^th^ cycle are used in this work. Table 3 shows that the optimum grid size for the subsequent simulations is 0.25 million cells (fine mesh). Considering both the computational time and accuracy, the optimum time step size is found to be 0.002T.

**Figure 3:**
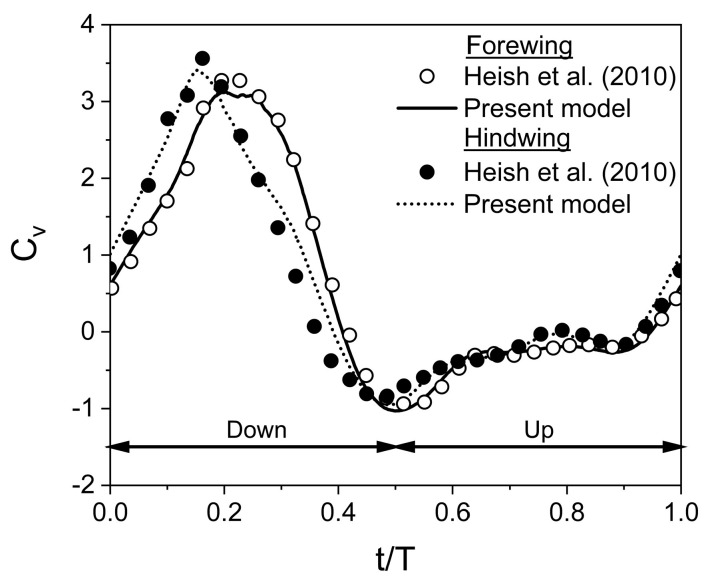
Validation of numerical model for a tandem flapping wing in inclined stroke plane [17].

To verify the validity of the adopted numerical model and wing kinematics, a 2D test case, similar to the study of Hsieh et al. [17], is simulated to reveal the aerodynamic characteristics of a tandem flapping wing (t/c = 10%) in inclined stroke-plane hovering. The wing kinematics used for the simulation are A_o_/c = 2.5, β = 60°, α_o_ = 45°, B = 45°, φ = 0°, ψ = 0°, f = 40 Hz, L/c = 1.2, Re = 625, and D/c = ∞ (no ground presence). Figure 3 shows that comparison of instantaneous C_v_ (after becoming stable or fully periodic) computed from the present study in comparison with Hsieh et al. [17]. An excellent agreement is observed between the present study and Hsieh et al. [17], confirming the numerical model’s validity.

**Figure 4:**
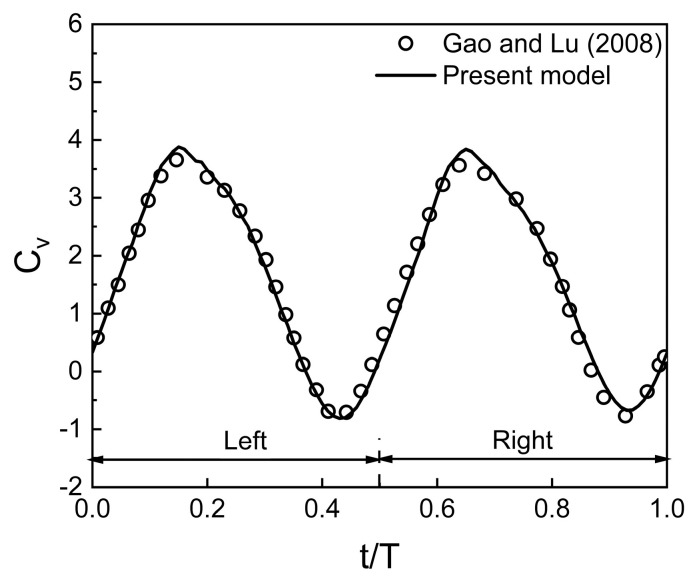
Validation of present model for a normal hovering flapping wing in the close proximity of the ground [25].

A second test case reported by Gao and Lu [25] is simulated using the present numerical model to verify its reliability in predicting the aerodynamic behavior of a normal hovering flapping wing in close proximity to a ground. The wing kinematics used for the tandem flapping wing are A_o_/c = 2.5, β = 0° (for a normal hovering), α_o_ = 90°, B = 45°, φ = 0°, Re = 100, and D/c = 1. The time histories of the vertical force coefficient are compared in Figure 4. For a wing in close proximity to the ground, the time histories of the vertical force coefficient obtained from the present work have good agreement with that of Gao and Lu [25].

**Figure 5:**
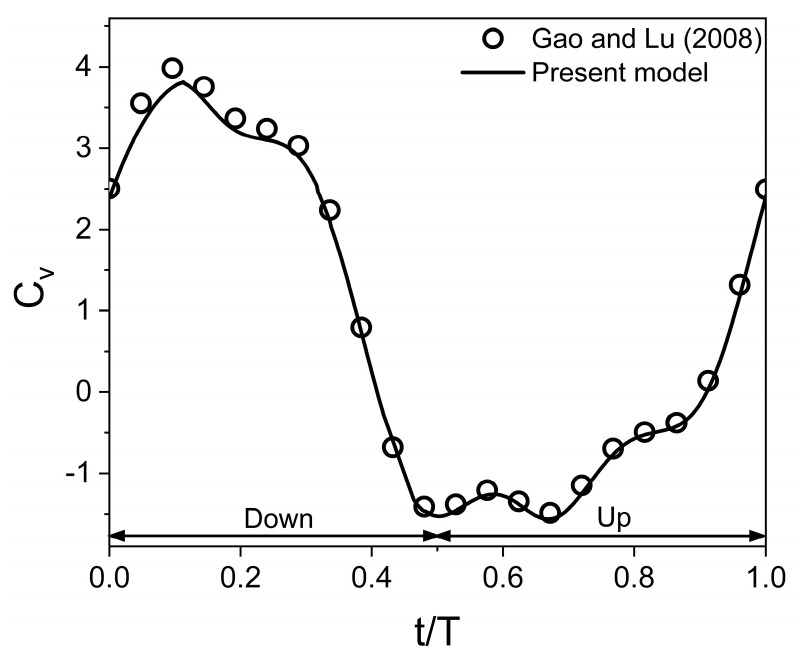
Comparison of C_v_ for a flapping wing in inclined stroke plane hovering [25].

A third validation study is performed to compare the results obtained from the present numerical model with Gao and Lu [25]. The wing kinematics used for a flapping wing in inclined stroke plane hovering are A_o_/c = 2.5, β = 60°, α_o_ = 45°, B = 45°, φ = 0°, and Re = 157. A good agreement is observed between the compared data of C_v_, as shown in Figure 5. The result from the present model is close to the data of Gao and Lu [25] for inclined stroke plane hovering kinematics as well.

**Figure 6:**
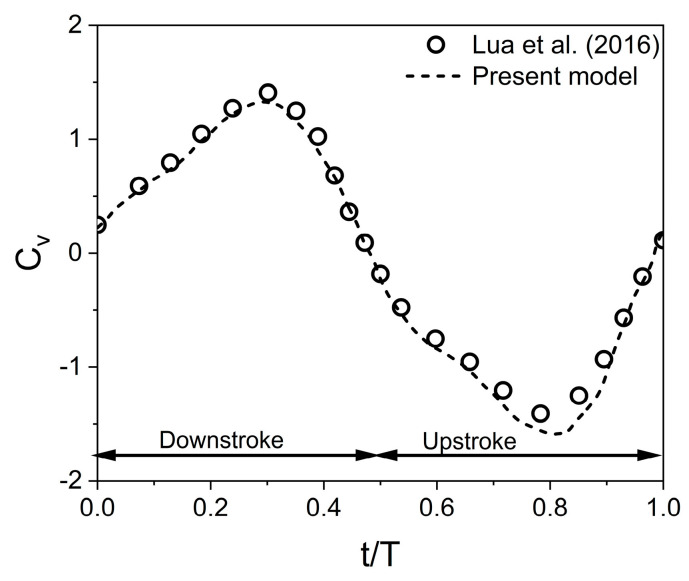
Comparison of C_v_ from numerical model with experimental results [43].

Due to the lack of experimental data on hovering tandem wings at low Re, a validation study compared the numerical model with the experimental results of Lua et al. [43] for a tandem flapping wing in forward flight at Re = 5000. While Broering and Lian [18] assumed laminar flow for tandem wings at this Re, turbulence modeling is enabled exclusively for this validation case. Following Lua et al. [43], the Spalart–Allmaras (S-A) model is employed to account for high Re effects. The other simulation parameters are A_o_/c = 1.5, β = 90°, f = 0.67 Hz, and Strouhal number St = 0.32. As shown in Figure 6, the present model closely aligns with the experimental data of Lua et al. [43], confirming its validity. However, some discrepancies may arise with the real-world performance due to three-dimensional effects.

## 3. Results and Discussion

The aerodynamic characteristics of a tandem flapping wing in ground proximity depend on both wing kinematics and flow condition. A total of 50 cases are simulated in this study examining the effect of various parameters of wing kinematics including flapping frequency f, stroke amplitude A_o_/c, and flapping patterns (in-phase and counter) in combination with ground distance D*. The dependence of aerodynamic force generation on viscosity and Reynolds number Re is also investigated.

### 3.1. Comparison of Aerodynamic Force Generation at Various Flapping Frequencies f

It is well known that insects like dragonflies mainly generate vertical force to balance the weight in the hovering flight. Insects like dragonflies dynamically adjust their stroke plane and body inclination to nullify the horizontal force generation in hovering flight [32,44]. Hence, some studies on dragonfly-based flapping wings have neglected horizontal force in their calculation for hovering flight [32,45]. However, the flapping wing kinematics in inclined stroke plane hovering is often modeled mathematically using simplified sinusoidal functions. As a result of this model, a net horizontal force is generated due to the difference in the horizontal force generation between the downstroke and the upstroke. Several past studies have reported on the horizontal force generation for a flapping wing in inclined stroke plane hovering when the wing kinematics is modeled using sinusoidal functions [9,12,17,39]. In this work, the horizontal force generation is reported, following previous studies [9,12,17,39].

**Figure 7:**
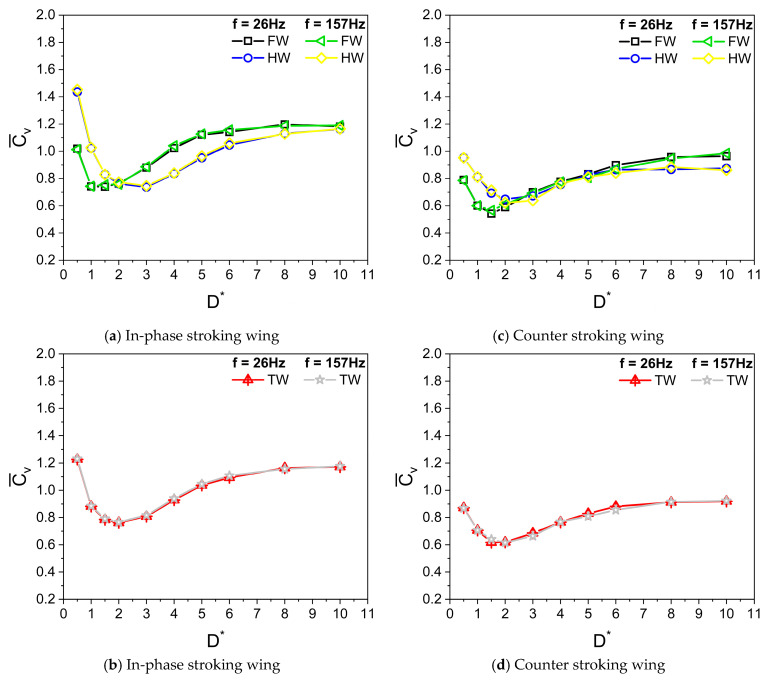
Variation of $\bar{C_{v}}$ with D* for in-phase and counter stroking flapping wings at different flapping frequencies f. (Fore wing, hind wing, and tandem wing are simplified by the terms FW, HW, and TW, respectively).

Dragonflies flap their wings with a frequency f in the range of 20 to 157 Hz. The average flapping frequency is estimated as 26 Hz [10,11]. The wing kinematics parameters used in the simulation are A_o_/c = 2.5, β = 60°, α_o_ = 45°, B = 45°, φ = 0°, and Re = 628. The distance D is measured at a time instance wherein the flapping wing reaches its bottommost position, in close proximity to the ground, as shown in Figure 2. Figure 7 illustrates the variation of average vertical force coefficient $\bar{C_{v}}$ with ground distance D* (D/c) for in-phase stroking and counter stroking, respectively, at different flapping frequencies f. The average force coefficients of the tandem wing are calculated by averaging the forewing and the hindwing.

It can be seen from Figure 7 that the flapping frequency f has no impact on the vertical force generation when the Reynolds number Re remains the same. For both in-phase stroking wings in Figure 7a,b and counter stroking wing in Figure 7c,d, as the ground distance D* increases, the $\bar{C_{v}}$ initially decreases to attain a minimum value and then recovers, eventually becoming constant beyond D* > 8. As shown in Figure 7a, the ground presence has beneficial effects on vertical force generation for an in-phase stroking wing at Re = 628 when the wing is in very close proximity to the ground (D* ≤ 0.5). The increase in the vertical force generation of the hindwing in Figure 7a could be primarily due to the vortex interaction between the forewing and the hindwing and the ground. In contrast, the beneficial effect of ground in vertical force generation at close proximity is not observed for a counter-stroking wing, as shown in Figure 7d.

The variations of $\bar{C_{v}}$ with D* in Figure 7 align well with the previous findings of Gao and Lu [25], in which normal force generation in normal hovering insects, such as fruit flies, in the ground presence is categorized into three kinds: force enhancement, force reduction, and force recovery. These three kinds are also observed in inclined stroke plane hovering insects, such as dragonflies, in ground presence. However, a very different trend is observed in the force generation of a pitching wing in the ground presence, where the normal force decreases monotonously with increasing ground distance [46].

**Figure 8:**
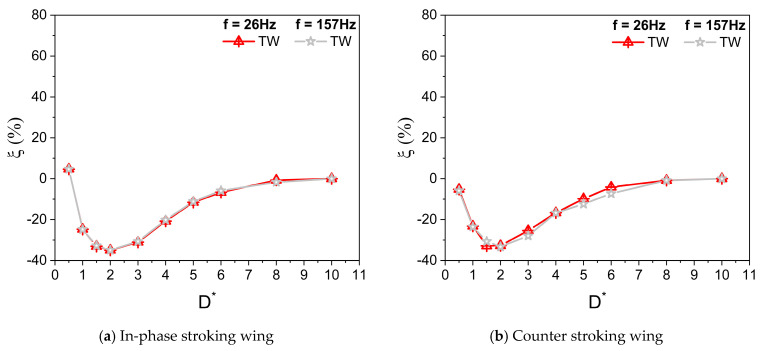
Vertical force enhancement factor ξ for in-phase and counter stroking flapping wings at different f. (Tandem Wing is simplified by term TW).

Figure 8a,b illustrate the vertical force enhancement, factor ξ, for in-phase and counter-stroking flapping wings at different f. For an in-phase stroking wing, a mild enhancement is observed in the vertical force due to the ground effect for D* ≤ 0.5, as shown in Figure 8a. For 1 ≤ D* ≤ 8, the wing ground interaction is primarily detrimental for vertical force generation, as shown in Figure 8a. In the case of a counter-stroking wing, the wing–ground interaction is always detrimental for vertical force generation at all D* values (0.5 ≤ D* ≤ 8), as shown in Figure 8b.

**Figure 9:**
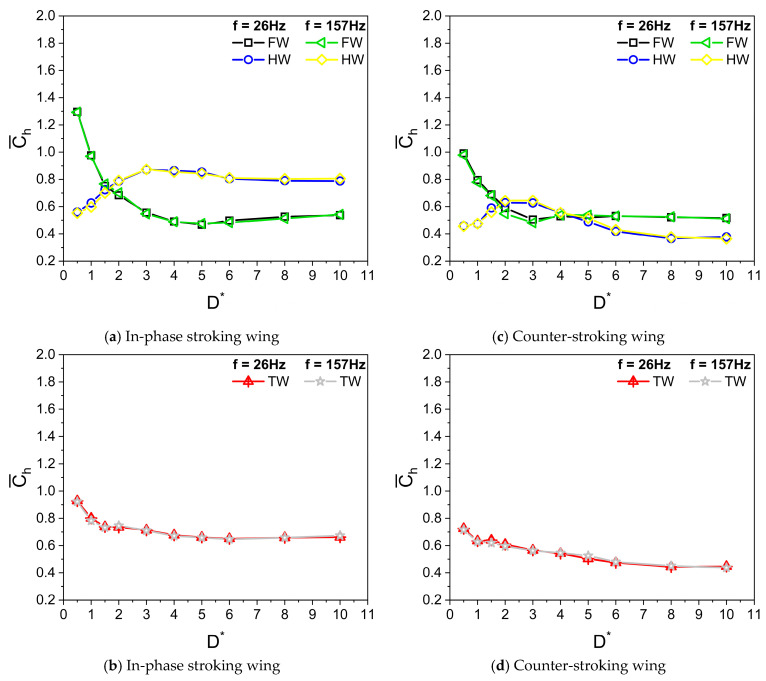
Variation of $\bar{C_{h}}$ with D* for in-phase and counter-stroking flapping wings at different flapping frequencies f. (Fore wing, hind wing, and tandem wing are simplified by the terms FW, HW, and TW, respectively).

**Figure 10:**
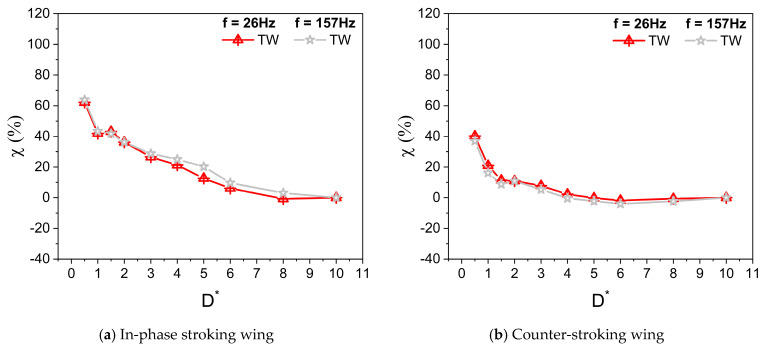
Horizontal force enhancement factor χ for in-phase and counter-stroking flapping wings at different f. (Tandem wing is simplified by the term TW).

Figure 9a,b and Figure 9c,d illustrate the variation of average horizontal force coefficient $\bar{C_{h}}$ with D* for in-phase stroking and counter stroking, respectively, at different f values. The $\bar{C_{h}}$ of the forewing gradually decreases and then becomes constant for D* > 6. On the contrary, the $\bar{C_{h}}$ of the hindwing first increases till D* = 3 and then decreases, eventually becoming constant for D* > 6. The ground presence is highly beneficial to the horizontal force generation for both in-phase and counter stroking wing at Re = 628, especially at smaller values of D*. A maximum enhancement of around 65% (when D* = 0.5) and 40% (when D* = 0.5) is observed in horizontal force generation for in-phase stroking and counter stroking, respectively, as shown in Figure 10a,b.

### 3.2. Comparison of Aerodynamic Force Generation at Various Stroke Amplitude A_o_/c

**Figure 11:**
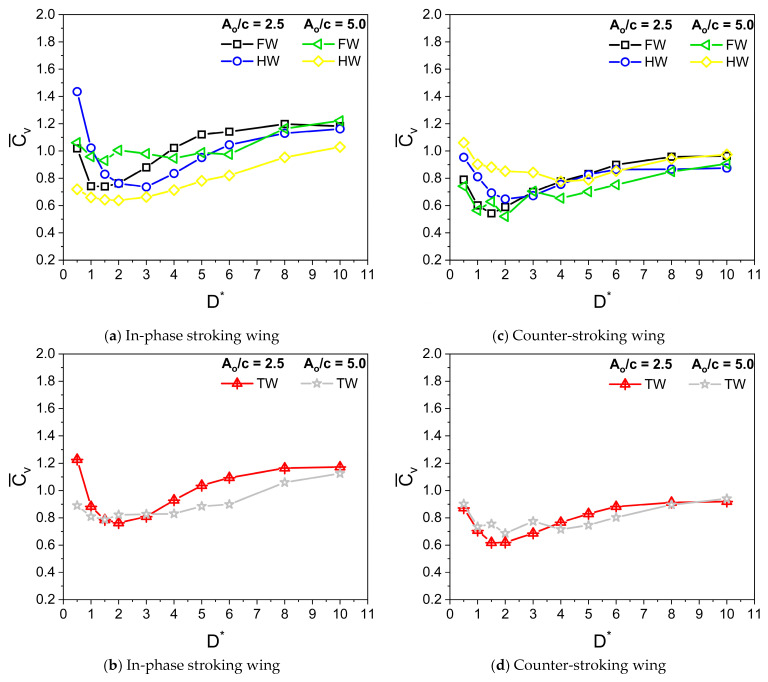
Variation of $\bar{C_{v}}$ with D* for in-phase and counter-stroking flapping wings at different stroke amplitude A_o_/c. (Fore wing, hind wing, and tandem wing are simplified by terms FW, HW, and TW, respectively).

**Figure 12:**
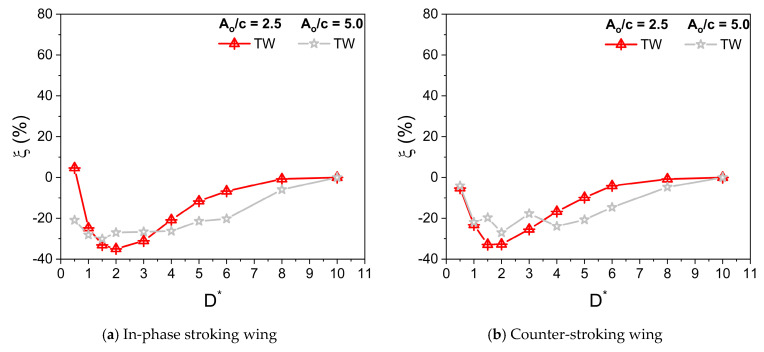
Vertical force enhancement factor ξ for in-phase and counter-stroking flapping wings at different stroke amplitude A_o_/c. (Tandem wing is simplified by the term TW).

Dragonflies flap their wings with stroke amplitude A_o_/c of 2.5–5 [8,9]. The parameters used in simulation are β = 60°, α_o_ = 45°, B = 45°, φ = 0°, and f = 26 Hz. Irrespective of A_o_/c, the Reynolds number Re is kept constant at 628. Figure 11 illustrates the variation of $\bar{C_{v}}$ for in-phase stroking and counter-stroking wings at different A_o_/c. At A_o_/c = 2.5, the ground effect is slightly beneficial toward the vertical force generation for an in-phase stroking wing at D* ≤ 0.5, as shown in Figure 11a,b. A maximum enhancement of around 5% is observed in the vertical force due to the ground presence, as shown in Figure 12a. However, at A_o_/c = 5, the benefits of ground effect on $\bar{C_{v}}$ is not observed for an in-phase stroking wing. The ground presence mainly causes detrimental effects on the vertical force generation of a counter-stroking wing for both stroke amplitudes, as shown in Figure 11c,d. This can be also inferred from the vertical force enhancement graph in Figure 12b.

**Figure 13:**
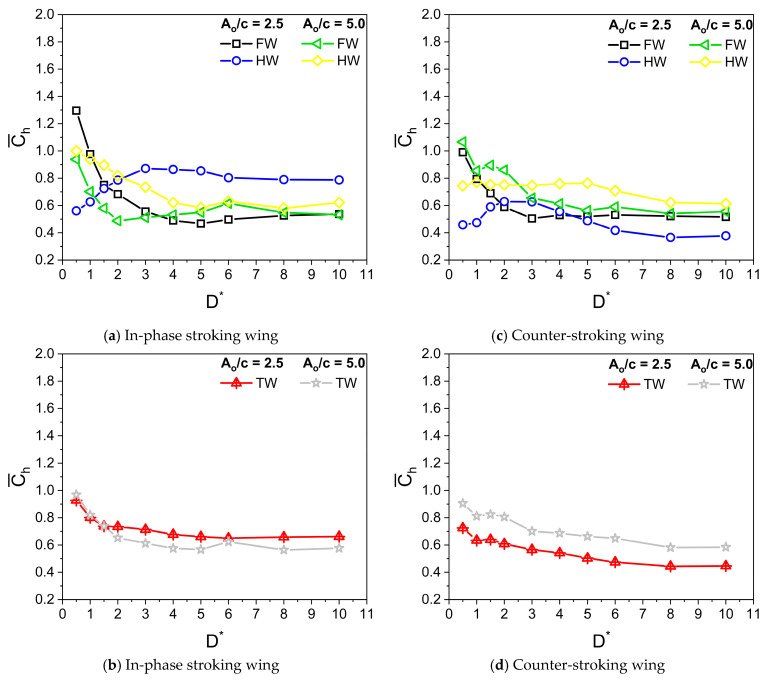
Variation of $\bar{C_{h}}$ with D* for in-phase and counter-stroking flapping wings at different stroke amplitude A_o_/c. (Fore wing, hind wing, and tandem wing are simplified by the terms FW, HW, and TW, respectively).

Figure 13 illustrates the variation of $\bar{C_{h}}$ for in-phase stroking and counter-stroking wings at different A_o_/c. At A_o_/c = 2.5 and 5, the ground effect is beneficial toward the horizontal force generation for both in-phase stroking and counter stroking wings for all D* values, as shown in Figure 13.

**Figure 14:**
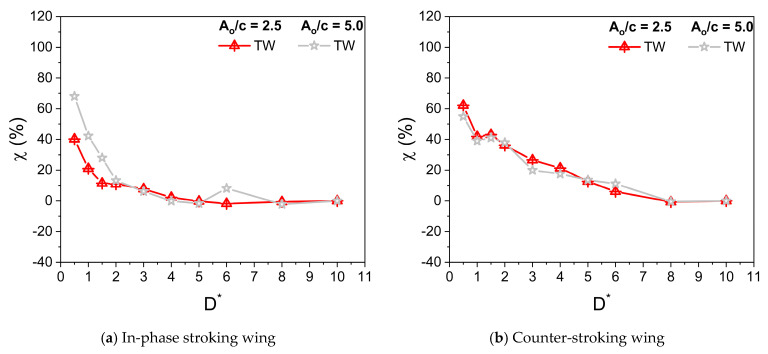
Horizontal force enhancement factor χ for in-phase and counter-stroking flapping wings at different A_o_/c. (Tandem wing is simplified by term TW).

A maximum horizontal force enhancement of around 70% is observed for the in-phase stroke wing due to the ground presence when A_o_/c = 5, as shown in Figure 14a. In the case of a counter-stroking wing, the maximum enhancement is around 60% when A_o_/c = 2.5, as shown in Figure 14b.

### 3.3. Comparison of Aerodynamic Force Generation at Various Reynolds Numbers Re

**Figure 15:**
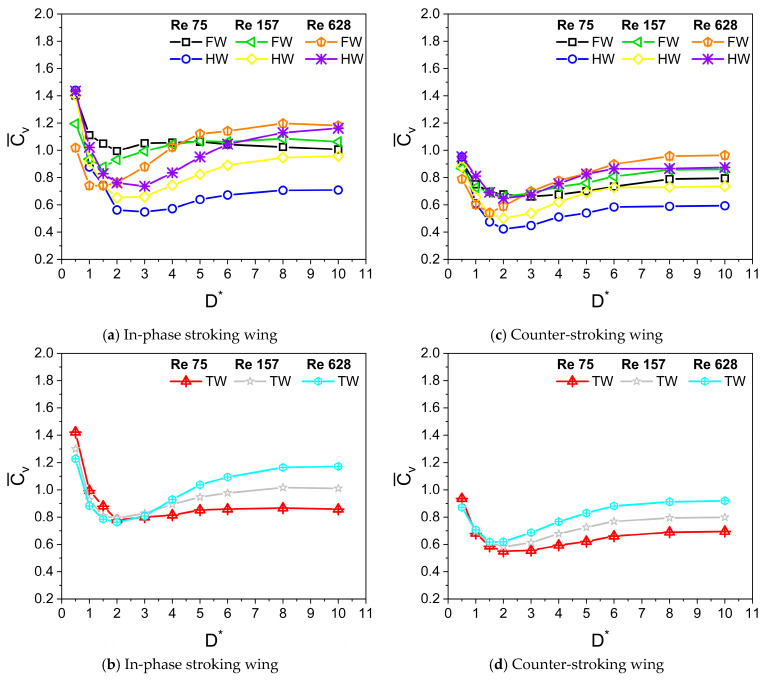
Variation of $\bar{C_{v}}$ with D* for in-phase and counter-stroking flapping wings at different Reynolds number Re. (Fore wing, hind wing, and tandem wing are simplified by the terms FW, HW, and TW, respectively).

The dependence of force generation on viscosity is studied in this section. The corresponding Reynolds number Re varies between 100 and 650, following Wang’s study [39]. For the results presented in this section, the wing kinematics parameters used for simulation are A_o_/c = 2.5, β = 60°, f = 26 Hz, α_o_ = 45°, B = 45°, and φ = 0°. The variation of $\bar{C_{v}}$ for in-phase stroking and counter-stroking wings at different Re is shown in Figure 15. Three different Re (Re = 75, 157, and 628) are used for performing the simulations. The forewing and hindwing contribution toward the vertical force generation can be inferred from Figure 15a. An interesting observation of very high vertical force is made for low Re cases when the tandem wing flaps in-phase in close proximity to the ground (D* = 0.5), primarily caused by the huge enhancement in the vertical force generation of the hindwing, as shown in Figure 15a,b. The reason behind this tremendous increase in vertical force is the complex interaction between the forewing, hindwing, and ground, which will be explained in the subsequent sections.

**Figure 16:**
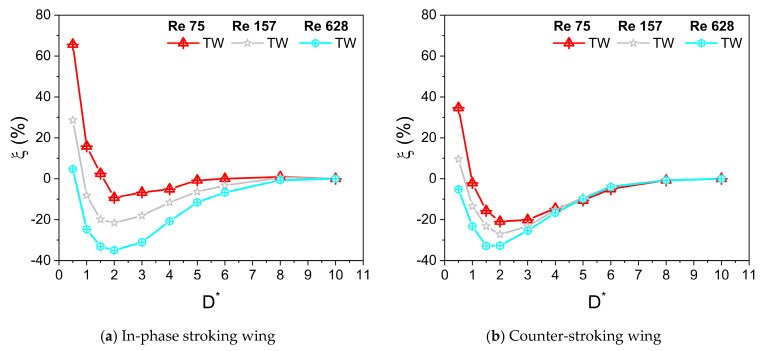
Vertical force enhancement factor ξ for in-phase and counter-stroking flapping wings at different Re. (Tandem wing is simplified by the term TW).

At Re = 628, the net vertical force of the in-phase stroking wing increases slightly (around 5% maximum enhancement at D* = 0.5) for ground distance D* ≤ 0.5 as compared to its away-from-ground case (D* = 10), as shown in Figure 15b and Figure 16a. As Re is decreased to 157, the net vertical force of the in-phase stroking wing increases moderately (around 10% maximum enhancement at D* = 0.5) for ground distance D* ≤ 1 as compared to its away-from-ground case (D* = 10). With a further decrease in Re (Re = 75), the net vertical force of the in-phase stroking wing increases tremendously (around 65% maximum enhancement at D* = 0.5) for D* ≤ 1.5 as compared to its away-from-ground case (D* = 10), as shown in Figure 15b and Figure 16a. The ground presence tremendously enhances the vertical force of the in-phase wing at close proximity to the ground (D* = 0.5) and also extends the favorable regime further at the low Re condition. A similar trend was observed in the vertical force generation of the counter-stroking wing. At low Re (Re = 75), the net vertical force generated by the counter-stroking wing increases tremendously (around 35% maximum enhancement at D* = 0.5) for D* ≤ 1 as compared to its away-from-ground case (D* = 10), as shown in Figure 15d and Figure 16b. Comparing the in-phase stroking and counter-stoking wing, it can be inferred that the vertical force generation of the in-phase stroking wing is always greater than the vertical force generation of the counter-stroking wing for all Re.

**Figure 17:**
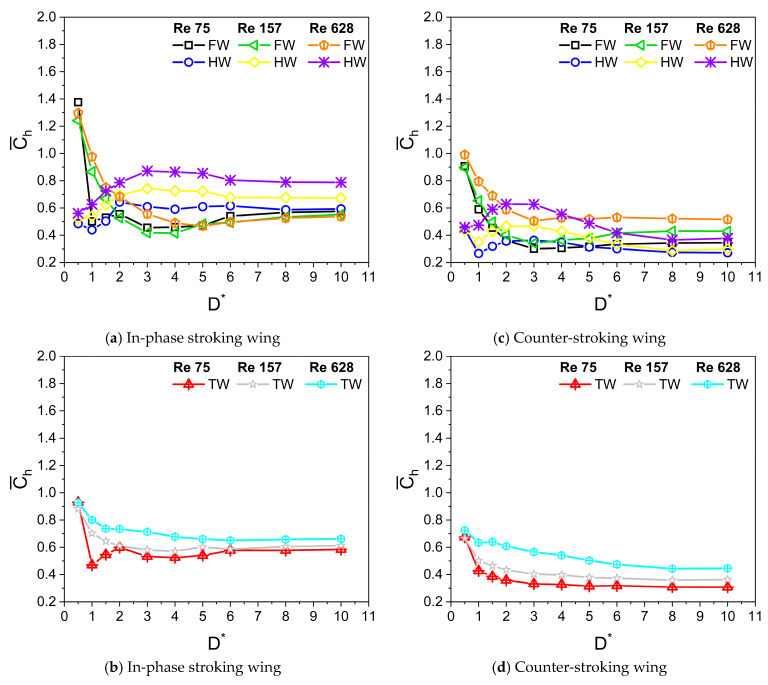
Variation of $\bar{C_{h}}$ with D* for in-phase and counter-stroking flapping wings at different Reynolds number Re. (Fore wing, hind wing, and tandem wing are simplified by the terms FW, HW, and TW, respectively).

**Figure 18:**
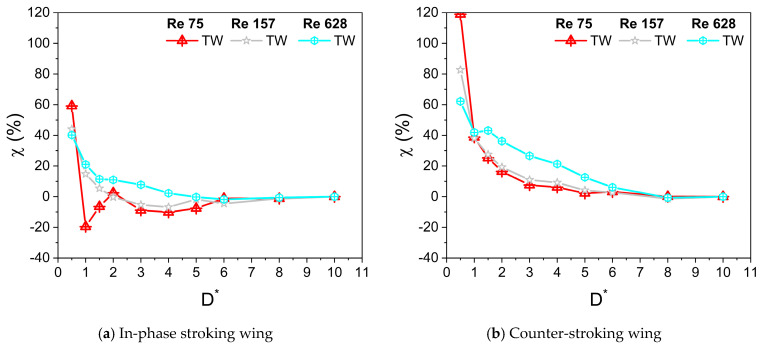
Horizontal force enhancement factor χ for in-phase and counter-stroking flapping wings at different Re. (Tandem wing is simplified by the term TW).

At Re = 628, the net horizontal force of the in-phase stroking wing increases significantly (around 40% maximum enhancement at D* = 0.5) for all D* as compared to its away-from-ground case (D* = 10), as shown in Figure 17b and Figure 18a. As Re is decreased to 157, the maximum enhancement in net horizontal force of the in-phase stroking wing is around 45% (at D* = 0.5) as compared to its away-from-ground case (D* = 10). At low Re (Re = 75), the net horizontal force of the in-phase stroking wing increases by around 60% (at D* = 0.5) as compared to its away-from-ground case (D* = 10), as shown in Figure 17b and Figure 18a. In general, the ground effect has a positive impact on horizontal force generation of in-phase wing at all Re.

In the case of a counter-stroking wing at Re = 628, the horizontal force generation increases tremendously for all D*, a maximum enhancement of 60% at D* = 0.5, as compared to its away-from-ground case (D* = 10), as shown in Figure 17d and Figure 18b. At Re = 157, the enhancement of horizontal force in the counter-stroking wing further maximizes around 85% at D* = 0.5. At low Re (Re = 75), the maximum enhancement in the net horizontal force generated by the counter-stroking wing is extremely high (around 120% at D* = 0.5) as compared to its away-from-ground case (D* = 10), as shown in Figure 17d and Figure 18b. Comparing the in-phase stroking and counter-stoking wing, it can be inferred that the horizontal force generation of the in-phase stroking wing is typically higher than the horizontal force generation of a counter-stroking wing for all Re.

### 3.4. Time Histories of Vertical and Horizontal Force Coefficients at Various Re

It is well known that unsteady mechanisms such as delayed stall, rotational lift, wake capture, and wing–wing interaction play a vital role in the vertical force generation of a tandem flapping wing in an inclined stroke plane hovering. These unsteady mechanisms are explained in detail in our previous studies using time histories of C_v_ and vortex structures for a tandem flapping wing in inclined stroke plane hovering without ground effect [16,45]. In the present study, the focus is mainly on the vortex interaction between the forewing, the hindwing, and the ground.

The tandem flapping wing motion in an inclined stroke plane hovering is shown in Figure 1. The definitions of rotational angle θ and angle of attack AOA are shown in Figure 1b and Table 1. For an in-phase stroking wing (ψ = 0°), the first half of a time period (t/T = 0–0.5) is a downstroke and the second half (t/T = 0.5–1) is an upstroke for both the forewing and the hindwing, as shown in Figure 1a. On the contrary, for the forewing of a counter-stroking wing (ψ = 180°), the first half of a time period (t/T = 0–0.5) is a downstroke and the second half (t/T = 0.5–1) is an upstroke. However, for the hindwing, the first half is an upstroke, and the second half is a downstroke, as shown in Figure 1b. During the downstroke, the wing moves with a high angle of attack AOA (AOA = θ), generating large vertical force by playing a key role in governing the leading-edge vortex. In the upstroke, the wing moves with a small angle of attack AOA (AOA = 180° − θ), generating a horizontal force (thrust) [47]. This asymmetry in AOA is not observed in normal hovering insects like fruit flies in which the stroke plane is horizontal. In normal hovering, the downstroke and upstroke have the same AOA [48].

**Figure 19:**
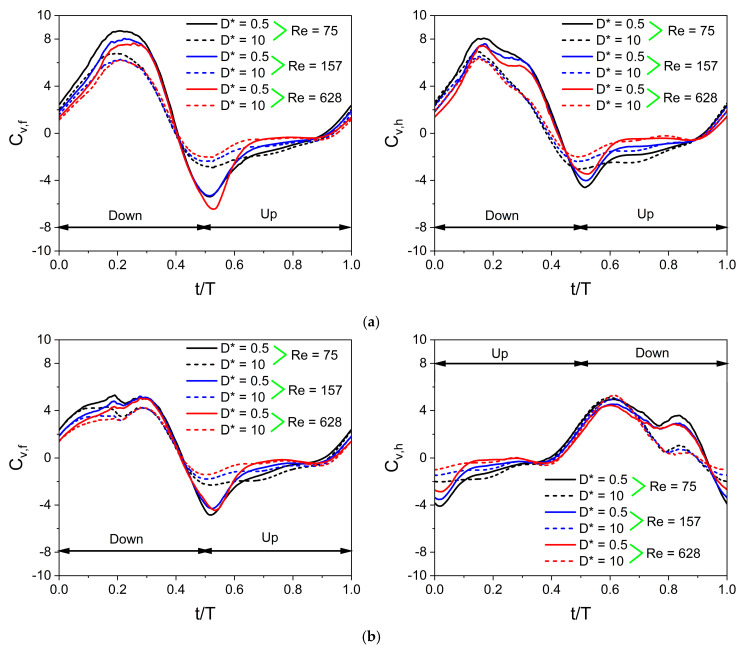
Instantaneous vertical force coefficient C_v_ for in-phase and counter-stroking flapping wings. (**a**) In-phase stroking wing. Forewing (**on the left**) and hindwing (**on the right**). (**b**) Counter-stroking wing. Forewing (**on the left**) and hindwing (**on the right**).

Figure 19 illustrates the time histories of C_v_ obtained at different Re. The parameters of wing kinematics used to simulate cases in Figure 19 are β = 60°, α_o_ = 45°, B = 45°, φ = 0°, A_o_/c = 2.5, and f = 26 Hz. Results of three different Re (Re = 75, 157, and 628) are presented. The variations in the local AOA during flapping motion significantly influence unsteady vertical force generation mechanisms. The cycle of the in-phase stroking wing starts with the time instant t/T = 0, where the instantaneous vertical force coefficient C_v_ is positive. The C_v_ of the in-phase stroking wing remains positive for the time intervals t/T = 0–0.4 and 0.9–1, whereas it is negative for the remaining interval. The time history of C_v_ for both forewing and hindwing exhibits one maximum (crest) and one minimum (trough) in a cycle, as shown in Figure 19a. Irrespective of D* and Re, the maximum C_v_ occurs at the middle of the downstroke for both wings, whereas the minimum C_v_ occurs at t/T = 0.5 when the wing reverses its stroke.

The vertical force generation for an in-phase stroking wing is shown in Figure 19a. In the case of D* = 0.5 and Re = 75, the C_v_ of the forewing gradually increases to reach a maximum (around 8.7) at the middle of the downstroke and then drops, as shown in Figure 19a. A similar variation is observed in the C_v_ of the hindwing, but its maximum C_v_ is slightly lower than the forewing, as shown in Figure 19a. In the case of D* = 10 and Re = 75, the maximum C_v_ for both forewing and hindwing decreases significantly. In the cases of D* = 0.5 and Re = 157, the maximum C_v_ of forewing and hindwing reduces moderately as compared to D* = 0.5 and Re = 75, as shown in Figure 19a. With a further increase in Re (Re = 628), there is only a slight reduction in the maximum C_v_ of forewing and hindwing.

As the forewing and the hindwing flap in-phase in the inclined stroke plane, it generates negative vertical force at the start of upstroke (t/T = 0.5), as shown in Figure 19a. However, the negative vertical force generated in the upstroke is significantly less than the positive vertical force generated in the downstroke. The negative C_v_ of both forewing and hindwing is also affected by D* and Re, as shown in Figure 19a. At D* = 10, the negative C_v_ at the start of upstroke (t/T = 0.5) reduces moderately for both wings even when the Re is the same. Meanwhile, as Re is increased from 75 to 157, the negative C_v_ at the start of upstroke (t/T = 0.5) reduces slightly even when D* is kept the same, as shown in Figure 19a.

The vertical force generation for a counter-stroking wing is shown in Figure 19b. In the case of D* = 0.5 and Re = 75, the C_v_ of the forewing gradually increases to reach a maximum (around 5.2) at the middle of the downstroke (t/T = 0.5) and then generates negative C_v_ in the upstroke, as shown in Figure 19b. Meanwhile, the hindwing initially generates negative C_v_ for the entire upstroke and then generates a maximum C_v_ (around 5) at the downstroke as shown in Figure 19b. Comparing in-phase stroking and counter-stroking wings, it is clear that the C_v_ of the in-phase stroking wing is significantly higher than the C_v_ of the counter-stroking wing, especially in the downstroke motion of both wings, as shown in Figure 19.

**Figure 20:**
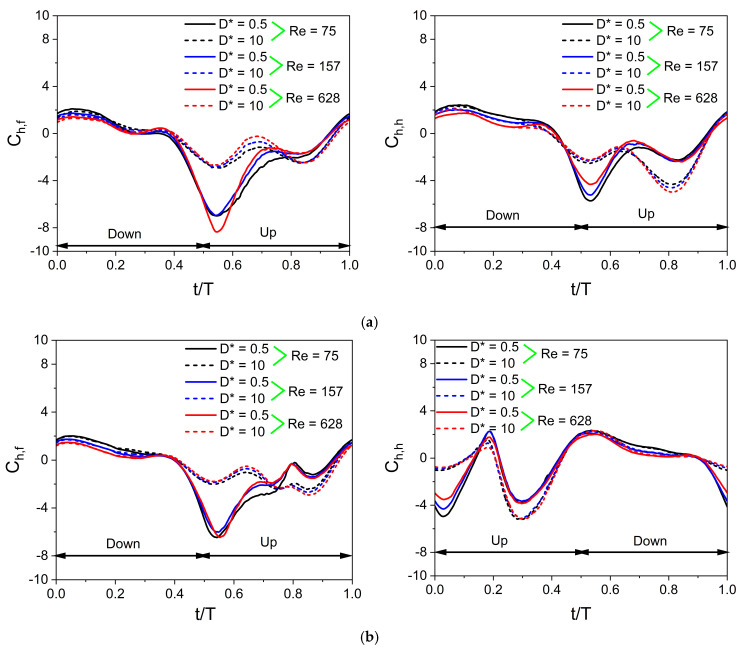
Instantaneous horizontal force coefficient C_h_ for in-phase and counter-stroking flapping wings. (**a**) In-phase stroking wing. Forewing (**on the left**) and hindwing (**on the right**). (**b**) Counter-stroking wing. Forewing (**on the left**) and hindwing (**on the right**).

The value of $\bar{C_{h}}$ is non-zero in the case of the inclined stroke plane hovering observed in dragonflies. If $\bar{C_{h}}$ is positive, it indicates the time-average drag force coefficient, and if $\bar{C_{h}}$ is negative, it indicates the time-average thrust force coefficient. Figure 20 illustrates the time histories of C_h_ obtained at different Re for in-phase stroking and counter-stroking wings. The cycle of the in-phase stroking wing starts with the time instant t/T = 0, where the instantaneous horizontal force coefficient C_h_ is positive for both forewing and hindwing, as shown in Figure 20a. The C_h_ of the in-phase stroking wing remains positive (drag) for the time intervals t/T = 0–0.4 and 0.9–1, whereas it is negative (thrust) for the remaining interval. It can be seen that most of the thrust force is generated when the flapping wing is in its upstroke.

In the case of an in-phase stroking wing, at D* = 0.5 and Re = 75, the forewing generates a very high negative C_h_ (around −7) in the upstroke, as shown in Figure 20a. A similar variation is observed in the C_h_ of the hindwing, but its maximum negative C_h_ is slightly lower than the forewing, as shown in Figure 20a. In the cases of D* = 10 and Re = 75, there are two peak values in the negative C_h_ of forewing (in the upstroke), which are of the nearly same magnitude, as shown in Figure 20a. The thrust generation of forewing decreases significantly for D* = 10, as is evident from the drastic drop in the maximum value of negative C_h_. In contrast, there are two peak values in the negative C_h_ of the hindwing (in the upstroke), which are of different magnitudes, as shown in Figure 20a. The occurrence of maximum negative C_h_ in the hindwing of the in-phase stroking wing at D* = 10 and Re = 75 is much delayed as compared to D* = 0.5 and Re = 75, as shown in Figure 20a.

The horizontal force generation for a counter-stroking wing is shown in Figure 20b. For cases with D* = 0.5, the maximum negative C_h_ in the forewing occurs at the start of the upstroke whereas there are two peak negative C_h_ in the hindwing that occurs at the start and middle of the upstroke, as shown in Figure 20b. Comparing in-phase stroking and counter-stroking wings, it is clear that the C_h_ of the in-phase stroking wing is moderately higher than the C_h_ of the counter-stroking wing, especially in the upstroke motion of both wings, as shown in Figure 20.

### 3.5. Vortex Structures and Surface Pressure Distribution

**Figure 21:**
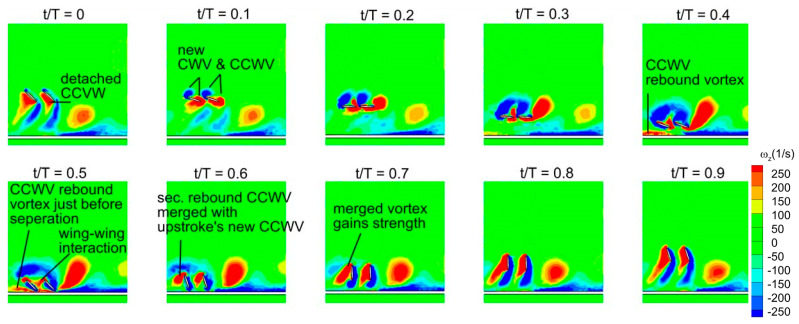
Vortex evolution of in-phase stroking flapping wings in ground effect.

To explain the effect of D* and Re on the vertical and horizontal force generation of in-phase and counter-stroking wings, the vortex structures and pressure graphs are plotted in this section. More focus is given to understand the mechanism of vertical force generation since it has a main role in the hovering flight. The vortex evolution of the in-phase stroking wing at D* = 0.5 and Re = 75 is illustrated in Figure 21. The time-histories of C_v_ in Figure 19a reveals that the vertical force generation of both forewing and hindwing gradually increases at the start of downstroke (t/T = 0–0.2) and then attains its peak at t/T = 0.2. Meanwhile, the vortex evolution in Figure 21 shows that there is a formation of a new vortex pair, a clockwise vortex (CWV) at the leading-edge and a counter-clockwise vortex (CCWV) at the trailing edge, on the upper surface of wings in the interval t/T = 0–0.2 for both wings. On the lower surface, the forewing and hindwing capture a detached CCWV.

In order to understand the existence of the detached CCWV on the lower surface, it is necessary to analyze the vortex structures during the stroke reversal period (t/T = 0.4–0.6) and in the upstroke. As forewing and hindwing changes from downstroke to upstroke (t/T = 0.4–0.6), a CCWV rebound vortex [32] forms due to ground presence, and subsequently, a part of it separates, giving rise to a CCWV secondary rebound vortex. This secondary vortex then merges with the new CCWV that forms at the start of upstroke in both forewing and hindwing. However, in case of hindwing, the contribution from wing–wing interaction also plays an important role since the CCWV (shown by red color in Figure 21) shed from the forewing fuses with the hindwing’s newly formed CCWV (at the start of upstroke), as clearly shown in t/T = 0.4–0.6. As the forewing and hindwing proceeds in the upstroke interval (t/T = 0.6–0.9), these merged vortices become stronger due to gradual growth of the newly formed CCWV in upstroke. In conclusion, it can be observed that the secondary rebound vortex caused by wing–ground interaction and wing–wing vortex interaction effects play a vital role in vertical force generation.

The effective angle of attack experienced by the wing depends on various factors such as the presence of vortices from previous stroke and local flow characteristics near the ground. From the obtained results and the findings of previous studies, the rebound vortex formed by the ground presence could change the effective angle of attack, which may increase or decrease the vertical force generation [32]. The rebound vortex and secondary vortex, caused by the rebound vortex, could also increase the effective angle of attack, which in turn aids the vertical force generation at small ground distance D* = 0.5.

The vortex evolution shown in Figure 21 also highlights the important role of stronger detached CCWV in generating enhanced vertical forces in inclined stroke plane hovering in the presence of the ground. This is different from normal hovering, where prior studies [25,49] suggest that the wake capture mechanism plays a key role in vertical force enhancement at smaller ground distances.

**Figure 22:**
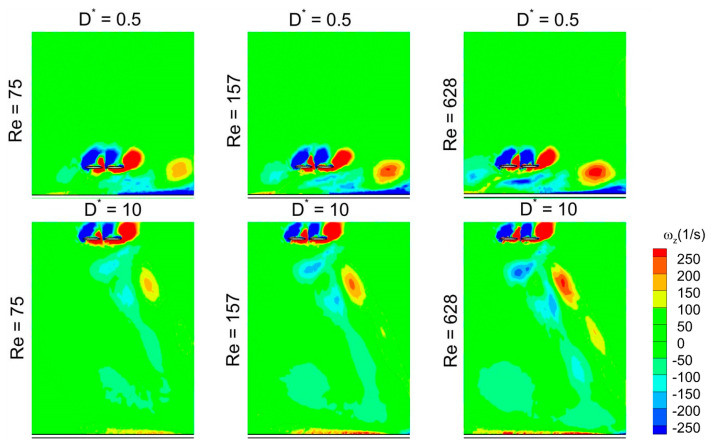
Effect of Re and D* on vortex structures of in-phase stroking flapping wings at t/T = 0.25.

**Figure 23:**
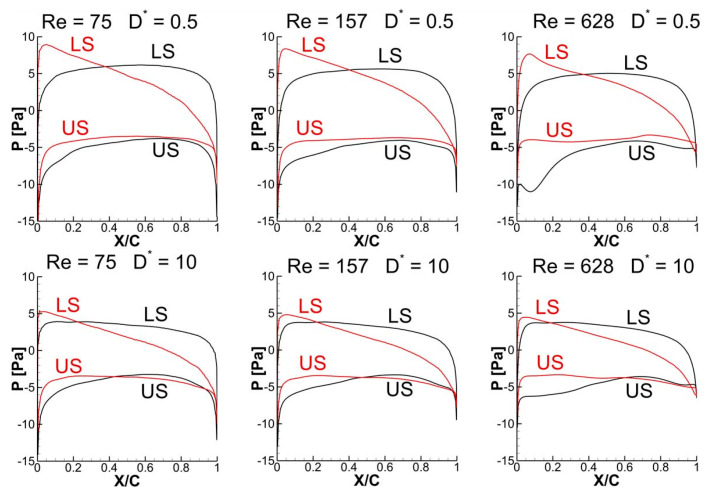
Effect of Re and D* on surface pressure distribution of in-phase stroking flapping wings at t/T = 0.25.

It is evident from Figure 22 that the detached vortices (on the lower surface) are strengthened by the strong CCWV secondary rebound vortex formed as a result of close proximity to the ground (D* = 0.5) as compared to D* = 10 for Re = 75 when the wing stays horizontal. Meanwhile, the surface pressure distribution at t/T = 0.25 in Figure 23 reveals that the wing–ground vortex interaction causes a larger pressure difference for D* = 0.5 as compared to D* = 10, resulting in more vertical force. As the Re is increased to 157 and 628, a slight drop is noticed in the vertical force, as shown in Figure 19a, compared to Re = 75. This is due to the reason that relatively weaker detached CCWV vortices are observed in Figure 22 for the cases Re = 157 and 628, compared to Re = 75. This is also evident from the pressure distribution plot in Figure 23 where the pressure difference is slightly lower for the cases Re = 157 and 628, compared to Re = 75. In conclusion, the wing–ground vortex interaction has a positive influence on the vertical force generation for the in-phase stroking wing.

As shown in Figure 1b and Figure 21, the forewing and hindwing of the in-phase stroking wing remain at a low AOA in the interval (t/T = 0.55–0.85), and therefore, the pressure difference between the upper and lower surfaces in that interval will primarily cause horizontal force generation. This can also be confirmed from the time histories of C_h_ for the in-phase stroking wing in Figure 20a. In this interval, both the forewing and hindwing are very close to the ground, as shown in Figure 21, which means the wing–ground interaction plays a huge role in both wings generating large horizontal forces. In conclusion, the wing–ground vortex interaction has a positive influence on the horizontal force generation for the in-phase stroking wing.

**Figure 24:**
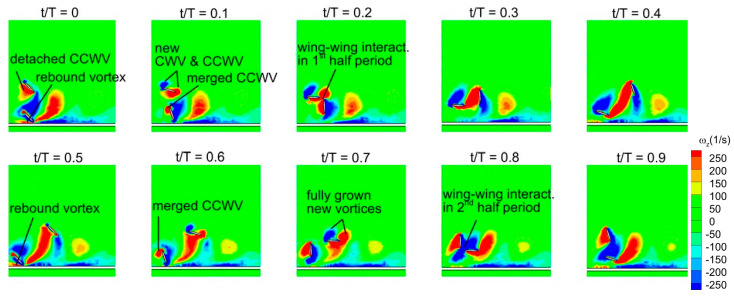
Vortex evolution of counter stroking flapping wings in ground effect.

The vortex evolution of counter stroking wing at D* = 0.5 and Re = 75 is illustrated in Figure 24. The time histories of C_v_ for a counter-stroking wing in Figure 19b show that the forewing generates the majority of vertical force in its downstroke interval (t/T = 0.1–0.35). Meanwhile, Figure 24 shows that a CWV vortex and a CCWV vortex are newly formed on the forewing’s upper surface. On the lower surface, a strong detached CCWV is observed. This strong detached CCWV exists as a result of merging the newly formed CCWV at the start of upstroke (t/T = 0.5–0.8) with the CCWV secondary rebound vortex formed as a result of ground presence at t/T = 0.5 during the stroke reversal. However, the wing–wing vortex interaction effect in the interval t/T = 0.2–0.3 does not play a constructive role in forewing’s vertical force generation due to a 180° phase difference. Therefore, the vertical force generation in counter stroking is not as high as in-phase stroking.

The hindwing (flapping in opposite with a 180° phase difference) generates the majority of vertical force in its downstroke interval (t/T = 0.55–0.85), as shown in Figure 19b. Similar to the forewing, a CWV vortex and CCWV vortex are newly formed on the upper surface. On the lower surface of the hindwing, a much stronger detached CCWV is observed. However, due to a 180° phase difference, the wing–wing vortex interaction effect in the interval t/T = 0.7–0.9 play a detrimental role in the vertical force generation. In conclusion, although wing–ground vortex interaction play a constructive role, the vertical force generation is much less in counter stroking mainly because of the negative contribution from wing–wing vortex interaction.

**Figure 25:**
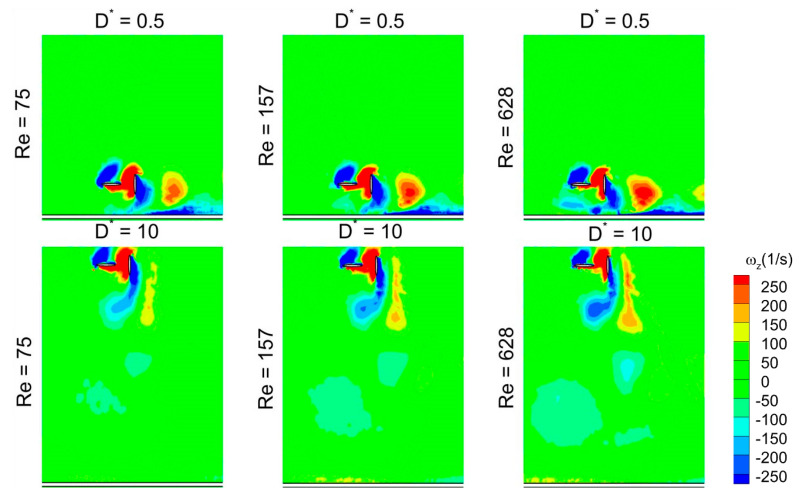
Effect of Re and D* on vortex structures of counter-stroking flapping wings at t/T = 0.25.

As discussed earlier, the detached vortices are strengthened by the strong CCWV secondary rebound vortex formed as a result of close proximity to the ground (D* = 0.5) as compared to D* = 10 for Re = 75 when the forewing stays horizontal at t/T = 0.25. This is the main reason behind the higher vertical force generation in the forewing of the counter-stroking pattern for D* = 0.5, compared to D* = 10, as shown in Figure 19b and Figure 25. However, for the hindwing, the vortex interaction between the forewing and the hindwing plays a crucial role in influencing the vertical force generation, especially in the interval t/T = 0.55–0.85, as shown in Figure 24. In this interval, the vertical force generation of the hindwing is increased by two mechanisms, wing–wing vortex interaction and wing–ground vortex interaction, as shown in Figure 19b and Figure 24. Furthermore, Figure 25 shows that the wing–ground vortex interaction strengthens the detached vortices of the forewing at D* = 0.5 compared to D* = 10 across all Re, resulting in higher vertical force generation. In conclusion, the wing–ground vortex interaction has a positive influence on the vertical force generation for the counter-stroking wing.

**Figure 26:**
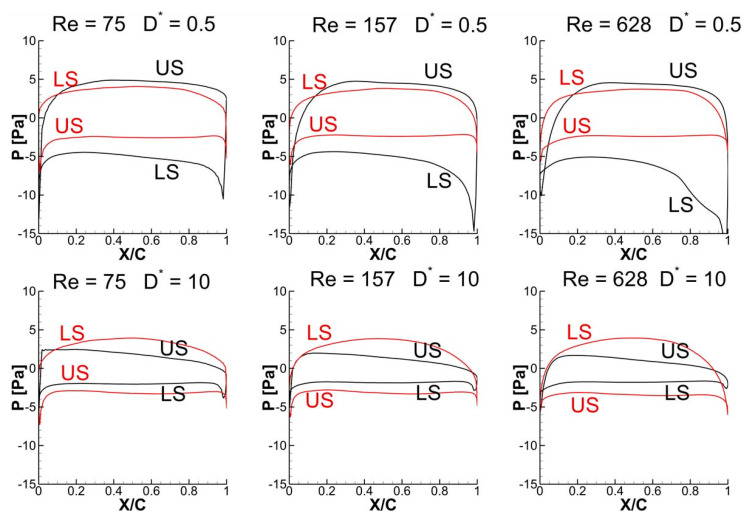
Effect of Re and D* on pressure distribution of counter-stroking wings at t/T = 0.55.

The time histories of C_h_ for a counter-stroking wing in Figure 20b show that the forewing stays at a relatively low AOA at its start of upstroke (t/T = 0.55) compared to the downstroke, and hence, the pressure difference between the upper and lower surfaces will result in horizontal force generation. At this instant, the forewing is very close to the ground for D* = 0.5 and the wing–ground vortex interaction influences the vortex evolution of the forewing, as shown in Figure 24. The effect of wing–ground vortex interaction on the horizontal force generation of forewing for D* = 0.5 can be explained with the help of the pressure plot shown in Figure 26. The wing–ground vortex interaction increases the pressure difference between the upper and lower surfaces in the forewing for D* = 0.5 at all flow Re, resulting in high C_h_ at t/T = 0.55 as compared to D* = 10. The wing–ground vortex interaction has a huge influence on the strength of newly formed CCWV and CWV vortices on the lower surface of the forewing, and thereby, large negative pressure is generated, as shown in Figure 24 and Figure 26. On the other hand, it has some influence on the detached CWV on the upper surface. In the case of D* = 10, the effect of wing–ground vortex interaction subsides, and hence, both the pressure difference and C_h_ are smaller. Meanwhile, the hindwing generates a large C_h_ at the start of its upstroke (t/T = 0.1) when it is very close to the ground due to the wing–ground vortex interaction, as shown in Figure 20b and Figure 24. In conclusion, the wing–ground vortex interaction has a positive influence on the horizontal force generation for counter-stroking wings.

### 3.6. Time Histories of C_v_ and Vortex Structures for a Wide Range of D*

The variations of $\bar{C_{v}}$ with D* in Figure 15 closely align with the findings of Gao and Lu [25]. In hovering flight, vertical force generation is crucial to balance weight. Therefore, the force behavior is categorized based on the vertical force shown in Figure 15b,d. Three distinct types are observed in vertical force: force enhancement, force reduction, and force recovery. For in-phase stroking at Re = 75, the force enhancement, force reduction, and force recovery occur when D* ≤ 1.5, D* = 2, and 3 ≤ D* ≤ 5, respectively. For counter stroking at Re = 75, the force enhancement, force reduction, and force recovery occur when D* ≤ 1, 1.5 ≤ D* ≤ 2, and 3 ≤ D* ≤ 6, respectively.

**Figure 27:**
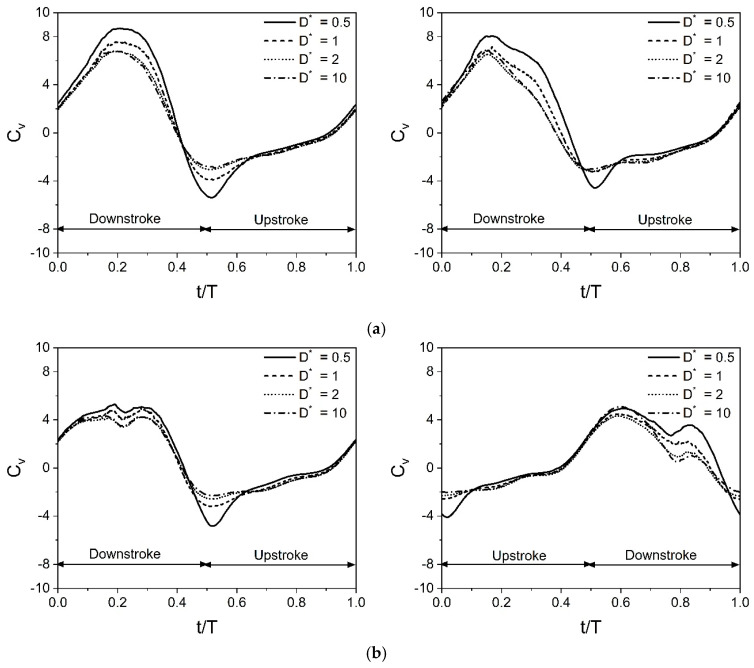
Instantaneous C_v_ of in-phase and counter-stroking wings for a wide range of ground distance D* at Re = 75. (**a**) In-phase stroking wing. Forewing (**on the left**) and hindwing (**on the right**). (**b**) Counter-stroking wing. Forewing (**on the left**) and hindwing (**on the right**).

**Figure 28:**
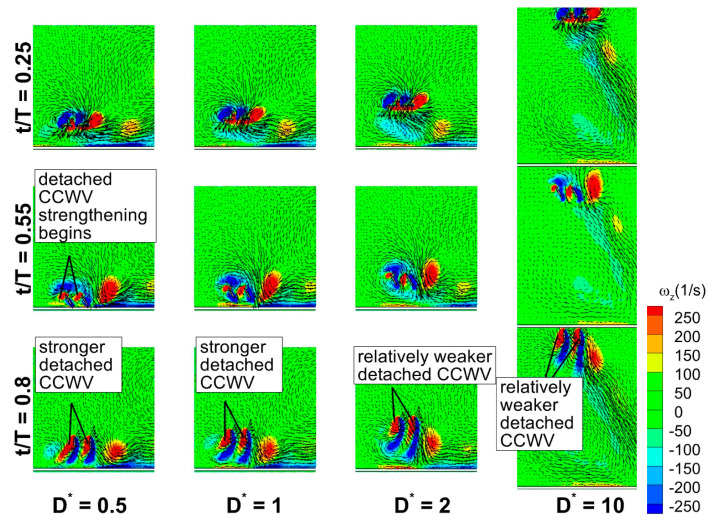
Vortex evolution of in-phase stroking wings for a wide range of ground distance D* at Re = 75.

Figure 27a shows the time histories of C_v_ for a wide range of D* (0.5, 1, 2, and 10) at Re = 75 for in-phase stroking wings. Among the considered cases of in-phase stroking, D* = 0.5 and 1 fall under the force enhancement type, while D* = 2 corresponds to a mild force reduction. The D* = 10 case corresponds to a fully recovered situation. As seen in Figure 27a, the peak C_v_ (crest) at the middle of a downstroke gradually decreases as D* increases from 0.5 to 10 for both the forewing and hindwing. At D* = 0.5 and 1, the close proximity of ground results in the detached CCWV being strengthened by the secondary rebound vortex at t/T = 0.25, as shown in Figure 28. In addition, the rebound vortex caused by the interaction has a huge impact on the vortex evolution by changing the effective angle of attack (from the velocity vectors), which could also increase vertical force generation. The strengthening of detached CCWV by the secondary rebound vortex and the role of rebound vortex is explained with the help of upstroke’s vortex structures (t/T = 0.55 and 0.8) in Figure 28. The detached vortex is relatively stronger for D* = 0.5 and 1 than its counterparts at t/T = 0.55 and 0.8. However, at D* = 2 and 10, the influence of rebound vortex on the vortex evolution diminishes, as evident from velocity vectors, resulting in substantial drop in vertical force generation.

**Figure 29:**
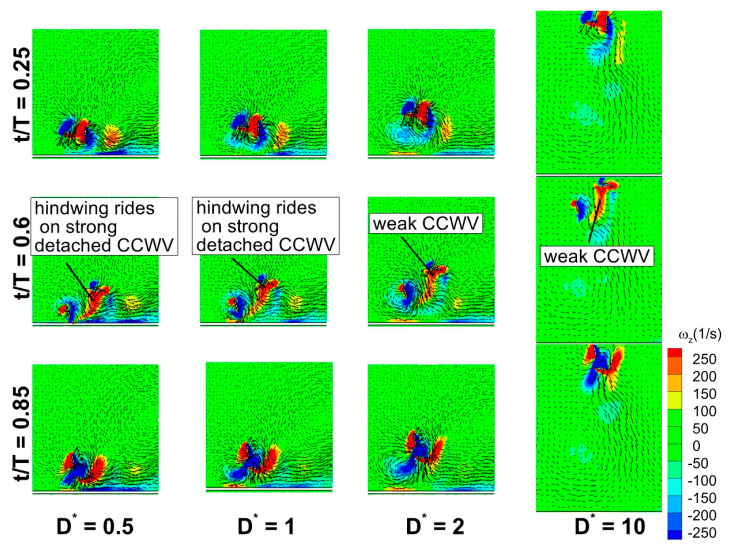
Vortex evolution of counter-stroking wings for a wide range of ground distance D* at Re = 75.

Figure 27b presents the time histories of C_v_ for a wide range of D* (0.5, 1, 2, and 10) at Re = 75 for counter stroking wings. Among these cases, D* = 0.5 and 1 correspond to force enhancement, while D* = 2 exhibits a moderate force reduction. The case of D* = 10 corresponds to a fully recovered situation. As observed in Figure 27b, the hindwing’s C_v_ of all cases has two distinct peaks at t/T = 0.6 and 0.85. An analysis of the forewing’s vortex structures in Figure 29 reveals a stronger detached CCWV at D* = 0.5 and 1 compared to D* = 2 and 10. Meanwhile, during its downstroke (at t/T = 0.6 and 0.85), the hindwing of cases D* = 0.5 and 1 rides on a relatively stronger detached CCWV, as shown in Figure 29. This flow feature explains the increase in vertical force for the hindwing within the interval t/T = 0.6–0.85 at D* = 0.5 and 1, compared to D* = 2 and 10.

## 4. Conclusions

The present study investigates the ground effect on the aerodynamic characteristics of a tandem flapping wing in inclined stroke plane hovering. The present two-dimensional simulations investigate the role of various parameters, including flapping frequency f, stroke amplitude A_o_/c, and flapping patterns (in-phase and counter), in combination with ground distance D* (D/c). The dependence of aerodynamic force generation on viscosity is also studied by varying the Reynolds number Re. The main findings of the study are as follows:

- The flapping frequency f has no effect on the vertical and horizontal force generation for both in-phase and counter-stroking patterns when the Reynolds number Re remain same.
- A large stroke amplitude A_o_/c decreases vertical force generation for both in-phase and counter-stroking patterns when the Re remain same.
- At low Re (Re = 75) or large viscosity condition, a tremendous increase in the vertical force is observed for in-phase stroking wings when D* is extremely small (D* = 0.5). A maximum vertical force enhancement of around 65% for in-phase pattern and 35% for counter-stroking pattern is observed when D* = 0.5, as compared to D* = 10. This enhancement primarily results from the strengthening of detached vortices on the lower surface of the wings at the middle of the downstroke when the ground distance is extremely small (D* = 0.5).
- The vertical force behavior of an inclined hovering wing in the presence of the ground exhibits similarities to the findings of Gao and Lu (2008), which were observed in a normal hovering wing in ground-effect. Three distinct types characterize the vertical force behavior of the inclined hovering wing: force enhancement, force reduction, and force recovery.
- The wing–wing interaction and secondary rebound vortex, caused by wing–ground interaction, play a key role in vertical force generation. The wing–ground vortex interaction has a positive influence on the vertical and horizontal force generation for the in-phase and counter-stroking wings.
- In general, the vertical and horizontal force generated by the in-phase stroking wing is greater than the counter-stroking wing across all studied Re.

## Figures and Tables

**Table 1:** Various wing motion parameters.

Parameter and the Source of Data	Value
Reynolds number Re|corresponding dynamic viscosity (kg/ms)	75|0.000333532 157|0.000159331 628|3.98327 × 10^−5^
Flapping frequency f [10,11]	26–157 Hz
Mean angle of attack α_o_	45°
Pitch amplitude B	45°
Phase difference between the translation and the rotation φ	0°
Stroke plane inclination β [8,9]	60°
Angle of attack AOA	Downstroke: θ° Upstroke: 180-θ°
Stroke amplitude A_o_/c [8,9]	2.5–5
Phase difference between the forewing and the hindwing ψ [17]	0° and 180°

**Table 2:** Pitch angles for all the stages.

Downstroke					
Stage	t/T	α°	Stage	t/T	α°
1	0	45	7	0.3	2.20
2	0.05	31.09	8	0.35	8.59
3	0.1	18.55	9	0.4	18.55
4	0.15	8.59	10	0.45	31.09
5	0.2	2.20	11	0.5	45
6	0.25	0			
**upstroke**					
**stage**	**t/T**	**α°**	**stage**	**t/T**	**α°**
11	0.5	45	17	0.8	87.80
12	0.55	58.91	18	0.85	81.41
13	0.6	71.45	19	0.9	71.45
14	0.65	81.41	20	0.95	58.91
15	0.7	87.80	21	1	45
16	0.75	90			

**Table 3:** Results of grid and time independence tests.

Grid Size (Million)	$\bar{C_{v,f}}$	$\bar{C_{v,h}}$
Medium (0.13)	1.150	1.083
Fine (0.25)	1.203	1.152
Refined (0.5)	1.209	1.161
**Time-Step Size (s)**	$\bar{C_{v,f}}$	$\bar{C_{v,h}}$
Medium (0.004T)	1.157	1.097
Fine (0.002T)	1.203	1.152
Refined (0.001T)	1.221	1.174

## Data Availability

The data presented in this study are available on request from the corresponding author.

## Abbreviations

The following abbreviations are used in this manuscript:

MAV	Micro-Aerial Vehicle
LEV	Leading-Edge Vortex
IB-LBM	Immersed Boundary-Lattice Boltzmann Method
NACA	National Advisory Committee for Aeronautics
AOA	Angle of Attack
PISO	Pressure Implicit with Split Operator algorithm
FW	Fore Wing
HW	Hind Wing
TW	Tandem Wing
CWV	Clock Wise Vortex
CCWV	Counter Clock Wise Vortex
